# Fear causes tears - Perineal injuries in home birth settings. A Swedish interview study

**DOI:** 10.1186/1471-2393-11-6

**Published:** 2011-01-18

**Authors:** Helena E Lindgren, Åsa Brink, Marie Klinberg-Allvin

**Affiliations:** 1 School of Health and Social Science, Dalarna University, Falun, Sweden; 2 Institute of Health and Care Sciences, Sahlgrenska Academy at the University of Gothenburg, Sweden

## Abstract

**Background:**

Perineal injury is a serious complication of vaginal delivery that has a severe impact on the quality of life of healthy women. The prevalence of perineal injuries among women who give birth in hospital has increased over the last decade, while it is lower among women who give birth at home. The aim of this study was to describe the practice of midwives in home birth settings with the focus on the occurrence of perineal injuries.

**Methods:**

Twenty midwives who had assisted home births for between one and 29 years were interviewed using an interview guide. The midwives also had experience of working in a hospital delivery ward. All the interviews were tape-recorded and transcribed. Content analysis was used.

**Results:**

The overall theme was "No rushing and tearing about", describing the midwives' focus on the natural process taking its time. The subcategories 1) preparing for the birth; 2) going along with the physiological process; 3) creating a sense of security; 4) the critical moment and 5) midwifery skills illuminate the management of labor as experienced by the midwives when assisting births at home.

**Conclusions:**

Midwives who assist women who give birth at home take many things into account in order to minimize the risk of complications during birth. Protection of the woman's perineum is an act of awareness that is not limited to the actual moment of the pushing phase but starts earlier, along with the communication between the midwife and the woman.

## Background

Perineal injuries and anal sphincter ruptures are serious complications of vaginal delivery. Most common consequences of perineal injury are pain and incontinence, which affect the quality of life of healthy women [1]. Other consequences identified are negative emotional and psychological effects on women's overall well-being [2]. The prevalence of anal sphincter rupture increased from 2.6 percent to 4.2 percent between 1994 and 2004, and approximately 3000 women sustain severe perineal injuries annually in Sweden [1]. A similar increase has been described in other European and other Nordic countries. Norway reports an increase from one percent in the 1960s to 4.3 percent in 2008. Finland, on the other hand, reports stable figures below one percent over the past decade [3].

Risk factors for anal sphincter rupture during delivery are described as nulliparity, high birth weight of the child, instrumental deliveries, episiotomy, adverse birth position, maternal age and epidural analgesia [4,5]. Women who were delivered in a semi-sitting position or who squatted during the pushing phase were at greater risk of sustaining perineal injuries [1]. It is further reported that prolonged labor significantly increased the risk for perineal injuries [5]. Avoidance of episiotomy has been identified as a protective factor in avoiding perineal injuries among first-time mothers. Forceps delivery caused anal sphincter ruptures more often than vacuum extraction and spontaneous delivery [6]. Instrumental deliveries and Cesarean section has become more prevalent over the past decade, and the prevalence of anal sphincter rupture in second and third births has consequently increased [7].

Lower frequency of perineal injuries and anal sphincter ruptures has been reported from studies of planned home births internationally [8-12]. A Swedish register study showed that women who gave birth at hospital had a five times higher prevalence of anal sphincter ruptures than low-risk women giving birth at home [13]. Women who choose to give birth at home differ from those who give birth in the hospital in some aspects: they have more children, have a higher level of education, smoke less and have a lower BMI [14]. However, after adjustment for these factors the differences in prevalence of perineal injuries remain.

Evidence indicates that the prevalence of perineal trauma is lower among women giving birth at home assisted by a midwife. It is not clear whether this has to do with differences in midwifery practice or other factors. There is a need to understand more about midwifery practice in home births settings in order to gain knowledge on how to protect women from perineal injuries.

## Methods

A qualitative approach, using open-ended qualitative interviews, was applied in this study. Individual interviews were conducted with twenty midwives in 2009. Information regarding the study was presented on the website "Föda hemma" (Giving birth at home) hosted by a network of midwives assisting women in home deliveries. Midwives interested in participating were contacted, informed about the purpose of the study and invited to take part. Eleven midwives first gave their consent to participate and another nine midwives were contacted through these eleven midwives and invited to be individually interviewed. The interviews took place in the participants' homes or at their workplace. The time taken for the interviews was between 30 and 75 minutes. All interviews were tape-recorded with the permission of the participants. Interview guidelines with open-ended questions were developed prior to the study (additional file 1).

The age range among the midwives was 31-62 years. The level of experience of women assisting home births varied from 1 to 29 years. The participants were domiciled in various parts of Sweden and they had experience of different areas of midwifery such as delivery wards, gynecological departments and perinatal health consultations.

### Analysis

Data analysis took place after data collection was completed. In this study we conducted qualitative content analysis using an inductive method, since there were no previous studies dealing with the phenomenon [15]. The inductive analysis is conducted with the aim to enhance understanding and to generate knowledge. When using content analysis it is assumed that, when classified into the same categories, words and phrases share the same meaning [16]. Prior to the analysis ÅB, HL and MKA read the total material several times. The data was divided into units of meaning that were condensed. The condensed units of meaning (consisting of either a single word or several sentences) were abstracted and labeled with a code by ÅB and HL independently. ÅB and HL then discussed the codes and diverging codes were re-evaluated and consensus was reached. Similar codes were grouped into sub-categories and subsequently also divided into main categories, critically questioned and compared within the research team. These categories were modified during the procedure to generate a broader and more subjective category system in order to capture the specifics in the data. Finally, a comparison between similarities and differences resulted in one main theme.

## Results

In the analysis of the midwives' experiences five categories and one overall theme emerged from the interviews. A description of the content of each category follows, illustrated by quotations from the text. The category system is presented in Table 1.

**Table 1:** Category system.

Sub-categories	Categories	Theme
Knowing the woman	Preparing for the birth	No rushing and tearing about
Knowledge of birth		
Making the room inviting		
	
No rushing	Going along with the physiological process	
Listening to the woman		
Identifying signs		
Being responsive		
	
Home environment	Creating a sense of security	
Communication Focus on the relationship		
	
Changing positions	The critical moment	
Guiding the woman		
Handling the pain		
	
Warm cloths Holding against baby's head Effects of the midwife	Midwifery skills	

### Preparing for the birth

The midwives stated that they prepared for the birth mentally and physically. They prepared by going through the woman's birth plan and her obstetric history and by collecting additional information from the couple. The midwives also took part in efforts to make the environment inviting and comfortable for the woman.

#### Knowing the woman

In a home birth the woman is known to the midwife ahead of the birth. The midwife has seen the woman at least once during pregnancy and talked to her about her expectations and fears. Talking about fear of tearing is described as one strategy in order to prevent this from happening. If the midwife is familiar with woman's fears she can be supportive during the birth. Knowing the woman prior to the birth is reported as reassuring for several midwives.

"It's easier to communicate when I know what the woman is afraid of and what her previous births were like. She also knows that she can trust me."

"Fear causes tears. When the woman is frightened her pelvic floor tightens and is more likely to tear. At home she usually finds the courage to resist the urge to push."

Knowing the woman prior to the birth was also described as contributing to the sense of security for the midwife. *"I feel much more insecure at the hospital as I don't know who I'm dealing with."*

#### Knowing about birth

The midwives describe how their previous experience from attending many births contributes to the way in which they handle their next birth. Previous experience of sphincter ruptures and severe perineal injuries gives them intuitive knowledge of when the perineum is in danger. They were aware of the risk of tearing. As one midwife put it:

"Maybe we cannot avoid it completely but we can always do our best to minimize the trauma."

Midwives who were more inexperienced referred to knowledge they had gained from older colleagues. Discussions with more experienced midwives about different strategies for handling labor in order to reduce the risk of tearing give them a sense of security.

"I had a very experienced midwife who was like a mentor to me. She once told me a story about the birth of a 5 kg baby; in the first birth the woman had had a sphincter rupture and this time she wanted to give birth at home. The midwife was very close to the woman and guided her through each millimeter during the pushing phase. Their collaboration saved the woman's perineum completely."

#### Making the room inviting

Women who have chosen to give birth at home often have an image of themselves giving birth in their own environment. The midwives get to know the women's expectations, and this helps them to make it easier for the woman to have her wish fulfilled. The midwives described this support as contributing to helping the woman relax.

"For one woman it was so important this time to give birth in the birthing pool. She was almost pushing but I could see that she was holding back, waiting for the water to fill up. We helped her into the pool and one minute later she gave birth without tearing at all."

When the midwife knows what is important to the woman she can assist her by bringing her the things she wants to be surrounded by or prepare for the birth by putting on music that the woman likes. All these things together make the woman feel comfortable and have confidence in her own ability, as described by the midwives;

"...if the woman feels calm and relaxed it makes her perineum relax and she feels that it will be alright, we have few lacerations at home..."

### Going along with the physiological process

All the midwives in the study described how hospital-based deliveries differed from home-based deliveries and their perception of what factors contributed to perineal trauma.

#### No rushing

Time and time limits were frequently mentioned by the midwives as a factor contributing to perineal trauma. The rushing of normal labor was considered to be the worst enemy of an uncomplicated birth. When they assisted home births they were still aware of time and attentive to any exceeding of the normal length of labor. They described time as less significant during a home birth, and there were other factors they paid more attention to at home.

"We do not think of watches as machines, but I would say that it is the one subject that really threatens normal birth and makes the women tear."

#### Listening to the woman

Instead of being aware of time the midwives describe how they listen to the woman. By following the woman in her labor and carefully doing what is needed during the different phases, the midwives avoid perineal trauma. They describe listening as being in contact with the woman and following her wishes.

"If you really care for a woman in labor you do what it takes to fulfill her wishes. The women usually know what feels best for them and when they don't know you can listen and make suggestions so that they can find out for themselves."

#### Identifying signs

Being attentive to any signs that signal stress or anxiety was described by the midwives as important in order to prevent perineal injuries. When assisting the woman at home the midwives report that they are able to be more observant to the individual and the normal process than when working at hospital.

"When I have a home birth I focus completely on the birth process and use all my skills to support the woman. The lack of technology helps me keep all my senses alert to every little sign she gives me."

One of the midwives told a story about a woman who was giving birth to her third child. It was the first home birth for the mother and previous births had ended with an episiotomy at the hospital. She describes the pushing phase:

"I was so aware of the perineum, I could almost feel the tension in my own body. The woman was on her hands and knees and she was really affected by the transition phase. I could see that she wasn't comfortable; she was more or less trying to escape from the situation. I suggested that she should lie on her side and started talking about completely different things as I wanted to move the focus away from the urge to push. She started laughing and relaxed until her baby started coming without any pushing at all."

#### Being responsive

One of the most important tasks in the home birth situation is to be able to follow the woman and at the same time be responsive to any changes during labor. As the birth proceeds the woman finds herself in states she could not anticipate and the midwives stress the importance of not getting stuck in any plans drawn up ahead of the birth

"Empowering the woman when she is about to give up, that's our job. This gives her the strength to go on with the contractions and use all her energy to make it go well."

Dealing with the times when the woman is about to lose confidence in her own ability was described by the midwives as significant for a successful birth. One midwife said that she actually carried the woman mentally when she felt that she was about to lose her grip. Supporting a woman who has confidence in her ability is not a major challenge but doing so when she is about to give up is considered to be one of the midwife's most important tasks. Distinguishing the situations when a woman needs a lot of support and when she can cope on her own is crucially important.

### Creating a sense of security

#### Home environment

Women who choose to give birth at home often imagine themselves giving birth in a chosen place in the house. The midwives describe how the level of preparedness and determination they see in women who give birth at home is rarely seen in hospitals.

"I think we are very much like animals, to feel secure we need some space of our own and I've seen many women making a "nest" ahead of the birth. The security of being there helps them through the birth"

#### Communication

Having good communication was also considered to be one of the most important factors in avoiding injuries during birth. During the pushing phase the midwives feel confident in knowing the woman and her needs. One midwife says that she often relates to what has been said ahead of the birth in order to remind the woman during pushing.

"I know that they don't remember anything when they are in the transition stage so I refer to things we have talked about during pregnancy and try to help the woman back to what she really wants."

Communication with the partner was also mentioned as important. He or she can sometimes provide the bridge between the woman and the midwife as the one who knows the birthing woman better than anyone else

"Once there was a woman who completely lost herself during transition. She cried like a baby and I couldn't reach her so I asked her husband to comfort her and hold her and tell her that he loved her. Then she calmed down and gave birth without suffering a tear."

#### Focus on the relationship

The midwives described home births as different from hospital births in many respects. One of these was that their attention was focused on the relationship between themselves and the couple and the relationship between the couple. Some of the midwives also stated that they focused on the relationship between the woman and the child that was about to be born.

"When I feel love between the parents and for the new life I feel more secure in the situation. This helps me be a better midwife and I think I handle the birth better."

### The critical moment

Crowning is the critical moment for tearing or not. The midwives reported that most of the job has to have been done at that point. There must be confidence in the relationship and communication must work smoothly so that all the attention can be focused on the arrival of the baby.

#### Birth positions

The midwives all mentioned birth positions as an explanation for injuries during birth. They stated that women who are free to choose the position they want for the birth usually find themselves in an upright position leaning forward;

"If I see that she finds it hard to relax I try to support her by suggesting a change, a woman who is pushing seldom thinks of this herself. I have never seen a woman who is comfortable on her back at this stage."

"You never know in advance how a woman is going to give birth but I always try to help her find the most comfortable position because she can then relax and her pelvic floor is more relaxed too."

#### Guiding the woman

Guiding during the pushing phase was described as important for first-time mothers. Gentle guiding as attention is focused on the progress of the baby's crowning was considered to help the woman avoid tearing. This could be done by praising her courage when she pushes but also by giving her support in withstanding the pressure from the baby's head without rushing.

"I breathe with the woman and encourage her to follow me if she doesn't do this by herself. For some women the pressure is so great that you need to lead them through this moment."

#### Handling the pain

The crowning of the baby's head is often experienced as the most painful part of the birth. In contrast to the contractions during the opening phase and the confusing sensations that might come with transition, crowning is pure pain. This phase is often critical, as described below.

"The woman sometimes wants to avoid the pain by pushing all she can. My aim is to help her handle the pain so that she can be in contact with her body and follow its signals. I do this by talking to her, touching her and encouraging her to hold on for a moment or two."

### Midwifery skills

The midwives in this study had had many years of training and told various stories of techniques and skills they had learnt along the way.

#### Warm cloths

Use of warm cloth to support the perineum and ease the pain during the birth of the baby's head was described. Blood circulation in the perineum is said to be facilitated by the warmth from the cloth. The midwives also say that women usually find that it relieves the pain.

"I assisted a woman who had asked for warm cloths during her previous delivery at hospital but was then told that they were of no use. She had a severe rupture in that birth and this time she was very anxious to have them. Why should we refuse a woman's request in this case?"

Using the cloths also helped the midwives to get an idea of the perineum and its elasticity. They consider this more helpful and in particular less painful than using their fingers to stretch the perineum from inside, a method that is widely used in hospital births.

"I feel sick every time I see a colleague stretch a woman's perineum with her fingers while the woman is having to cope with all the pain from inside due to the pressure. There is no reason to make something that is difficult even worse."

#### No touching

Some midwives do not touch the perineum at all unless the woman asks them to or they recognize a need to do so. One of the midwives used a mirror to show the woman what happens so that she could control her pushing. Others report that they encourage the woman to take control of the emergence of the baby's head. Sometimes this is done by suggesting to the woman that she should feel the process with her own hands.

"For first-time mothers it is really rewarding to feel the baby's head. The labor might have felt as though it would never end, and now she can feel with her own hands that it will soon be over."

"When the mother follows the delivery of the baby's head by putting her hand on the head she pushes the exact amount it takes to help the baby out without tearing."

#### Effects of the midwife

All the midwives believed that they had an impact on the outcome with regard to perineal injuries. Being communicative and having trust in the woman's ability were factors that were frequently reported. Being able to take action when the woman is lost in the birth process is another factor. Homebirth midwives usually work in pairs and this was seen as a protecting factor.

"When I feel unsure or confused I ask my colleague to go in for me. You need to be humble and accept that you're not on top of the situation all the time. I may be tired and discouraged during a birth but I always try to keep these feelings away from the woman."

## Discussion

This interview study aimed to describe how midwives handle home births with the focus on protecting the perineum from injuries, which are associated with long-term consequences for many women.

### Methodological considerations

The study was based on a small and purposive sample, and as a result may not be representative. The study nevertheless provides important insights into midwifery practice at home births and how they serve as protective factors in relation to perineal injuries. There are, however, a number of methodological considerations that need to be taken into account when interpreting and transferring the results derived through the use of qualitative methods. In qualitative interviews, the influence of the researcher during the interview, as well as during the process of analysis, needs to be taken in consideration [17]. Reflexivity refers to the researchers' preconceptions based on previous personal and professional experiences and on their values and beliefs in relation to the topic studied [18]. The interviewer was a midwife with experience from both hospital and home-based deliveries. All the authors took part in the process of analysis, and this together with a continuous dialogue between the members of the research team enhances credibility [15]. The degree of dependability is thought to be high due to well-organized and prepared interview guidelines provided by a person with prior knowledge of home-based deliveries and their context. We argue that the issue of transferability is met in this study by the clear description of the data collection and process of analysis.

## Discussion of results

The midwives reported that they prepared for the planned home birth mentally as well as physically. They also took part in making the environment inviting and comfortable for the woman. They described the preparatory phase as a factor that might contribute to the lower frequency of perineal trauma in home births. This is in good agreement with a large study exploring factors related to safety in a planned home birth, which found knowing the mother and her home environment to be important [19]. The intuitive knowledge that guides the midwife in her management is described as being facilitated by acquaintance from prior to the birth and also by the environment [20].

All midwives in our study had experience of both hospital and home-based deliveries and described how care and routines differed. The midwives in this study describe how the home birth environment itself had a positive effect on the women. They experience time and time limit in the hospital setting as one factor contributing to perineal injuries. The home environment allows the midwives to follow the normal birth process without rushing. Hospital routines and interventions are identified as risk factors in the light of their attitude towards birth [21]. Birthing women seem to find courage in the environment they have created. Murphy and Feinland [9] consider it plausible that midwives contribute to keeping the perineum intact and avoiding episiotomies in a certain setting that has been chosen by low-risk women.

Being attentive to any signs from the woman that signal stress or anxiety was described by the midwives as important in order to prevent perineal injuries. Guiding the women should not be confused with forcing her to push. Gentle guiding was considered to help the women avoid tearing. According to Sampselle and Hines [22], a spontaneous birth protects the perineum from tearing. Women who pushed on demand from the staff had a higher frequency of perineal injuries and episiotomies than those who pushed when they felt the physiological urge to do so. Laine et al. [3] suggests that slowing down the birth of the baby's head by placing a hand on the crown and actively instructing the mother not to push while the head is being born reduces the prevalence of anal sphincter ruptures by 70 percent. Few midwives seem to use this method during a homebirth.

Midwives included in this study who attend home births report that their focus is on the women's strengths rather than their weaknesses. They also emphasize the aspects of safety, harmony, awareness, and integrity. By following the women in labor and doing what is needed during the different phases the midwives believe that they avoid perineal trauma. The women give birth on their own terms and in accordance with their requests. This is in line with previous research describing midwives' attitudes towards their attendance at home births [20]. It has been shown that giving birth at home means preserved authority and autonomy through which women have faith in life itself and rely on natural forces. Women described faith in their own competence, they received support that was chosen personally and they were able to be at home. Women who give birth at home wish to have control during birth and state that they trust their own ability to give birth [23]. One finding of this study was the description of communication as an important asset for a positive outcome of a planned home birth. Trust is fundamental to the communication between the midwife and the woman. According to Brunstad, Nilsen and Aasheim [24], midwives perceive that both cooperation and communication are important in the birthing room. However, this was sometimes difficult for midwives working at the hospital since they did not know the woman before labor started. The Norwegian midwives felt that this could contribute to a lack of guidance and consequently more injuries [24].

There is little evidence regarding the relationship between birth position and perineal outcome. Shorten and Donsante [25] suggest that the choice of birth position might contribute to the outcome regarding perineal injuries. Kneeling positions do not increase the risk for anal sphincter rupture compared to a semi-sitting position [26]. The midwives in this study all mentioned adverse birth positions as a factor contributing to injuries during birth. They reported that women who are free to choose the position they want for the birth usually find themselves in an upright position leaning forward. It was also considered important for the midwife to let the birthing woman try different positions and then help her find something better. Lindgren [27] reports that the most frequently used birth position in planned home births was upright kneeling followed by crouching on all fours or standing up. Gottvall, Allebeck and Ekéus [1] conducted an observational cohort study to assess the role of birth positions in the occurrence of anal sphincter tears. Their main finding was that women who used lithotomy (woman in a semirecumbent posture with her legs in stirrups) or a squatting position in the second stage of labor had a higher risk for anal sphincter tears than women using other positions. It also reduced the woman's opportunities to be in control during the pushing phase. Evidence that helps women to make informed choices regarding birth positions and perineal trauma have been presented by Soong & Barnes [28]. Access to the midwife also has a bearing on the birth outcome [29]. In planned home births the midwife is responsible for one woman at a time and is therefore readily available for the woman in labor, which is not always the situation in the hospital setting.

## Conclusion

All the midwives in this study stated that they had influence on the outcome with regard to perineal injuries. Midwives who attend home births view the birth as a physiological process, which is best, handled by giving support without interfering with the woman's instinct. The attitude towards time and time limits was seen as the most significant difference between home and hospital births. Most women who give birth at home are healthy multiparas, well prepared for the alternative they have chosen and with support from their partner. This might enhance the prospects of an optimal outcome for the mother emotionally as well as physically in terms of a spontaneous vaginal delivery without interventions or perineal trauma. It is important to further disseminate the knowledge home-birth midwives possess regarding the birth process and also to provide women with adequate information regarding the forthcoming birth of their child.

## Competing interests

The authors declare that they have no competing interests.

## Authors' contributions

All three authors contributed to the study; the study was planned by HL and MKL, data were collected by HL and ÅB, analysis was performed by the whole group, and all three participated in the writing of the manuscript.

## Pre-publication history

The pre-publication history for this paper can be accessed here:

http://www.biomedcentral.com/1471-2393/11/6/prepub

## Supplementary Material

Additional file 1 **Interview guidelines**. Following guidelines were used during all interviews with the midwives. • Could you tell about your experience of planned homebirths? • Could you please describe your activities (if any) that you use in order to prevent perineal injuries? • What do you think is the explanation for lower frequency of perineal injuries in home birth settings? Is there anything else that you consider significant for giving birth with an intact perineum? Click here for file
