# Supplementary material for: Fear causes tears - Perineal injuries in home birth settings. A Swedish interview study
Source: BMC Pregnancy Childbirth. 2011 Jan 18;11:6. doi: 10.1186/1471-2393-11-6 (PMC3034711; doi:10.1186/1471-2393-11-6)
Supplement: Additional file 1 — Interview guidelines. Following guidelines were used during all interviews with the midwives. • Could you tell about your experience of planned homebirths? • Could you please describe your activities (if any) that you use in order to prevent perineal injuries? • What do you think is the explanation for lower frequency of perineal injuries in home birth settings? Is there anything else that you consider significant for giving birth with an intact perineum? [file 1471-2393-11-6-S1.DOC]

Interview guidelines

- Background; age, years in midwifery, place of work.
- Years of experience in home births.
- Could you please describe how you handle the second stage in a home birth?
- What do you think are the reasons for perineal injuries?
- Do you use any special techniques for preventing tears?
- Can you describe a birth in which you believe that the outcome regarding perineal tears would have been different if not… (explain what happened).
